# Enhancement of Heat Dissipation by Laser Micro Structuring for LED Module

**DOI:** 10.3390/polym10080886

**Published:** 2018-08-08

**Authors:** Libin Lu, Zhen Zhang, Yingchun Guan, Hongyu Zheng

**Affiliations:** 1 School of Mechanical Engineering & Automation, Beihang University, Beijing 100191, China; lulibin@buaa.edu.cn; 2 State Key Laboratory of Tribology and Institute of Manufacturing Engineering, Department of Mechanical Engineering, Tsinghua University, Beijing 100084, China; zzhang@tsinghua.edu.cn; 3 Beijing Key Lab of Precision/Ultra-Precision Manufacturing Equipment and Control, Tsinghua University, Beijing 100084, China; 4 National Engineering Laboratory of Additive Manufacturing for Large Metallic Components, Beihang University, Beijing 100191, China; 5 Hefei Innovation Research Institute of Beihang University, Hefei 230013, China; 6 School of Mechanical Engineering, Shandong University of Technology, Zibo 255000, China; Hy271004@gmail.com

**Keywords:** laser surface texturing, heat dissipation, heat sink, LED chip, flexible electronics

## Abstract

Optimization for heat dissipation plays a significant role in energy saving and high-efficiency utilizing of integrated electronics. In this paper, we present a study of micro structuring on polymer-based flexible substrate coupled with aluminum-alloy heat sink. The heat dissipation performance was investigated by temperature evolution of a heat sink under natural convection by infrared (IR) camera, and results showed that the heat dissipation enhancement could be up to 25%. Moreover, the heat dissipation performance of a typical heat sink in terms of light-emitting diode (LED) hip was investigated via both thermal transient measurement and the finite element analysis (FEA). The maximum LED chip temperature of the laser-textured heat sink was approximately 22.4% lower than that of the as-received heat sink. We propose that these properties accompanied with the simplicity of fabrication make laser surface texturing a promising candidate for on-chip thermal management applications in electronics.

## 1. Introduction

Heat dissipation has become an important challenge for rapid development of modern electronic devices due to energy consumption and utilizing performance [1,2,3,4,5]. New materials including carbon nanotubes [6,7], graphene quilts [8,9,10] and nanocomposites [11,12] with high thermal conductivity have been widely investigated. Unfortunately, the fabrication processing of these materials is complicated and expensive, thus limiting their practical application in electronics [13]. 

Surface structures have been considered as an effective way to enhance heat transfer properties under natural convection [7,14]. Lee et al. [15] used a conventional mechanical machine to fabricate micro-channels with hydraulic diameters ranging from 318 to 903 μm on copper, and indicated that the heat transfer coefficient was inverse proportional to channel size at a given flow rate. Yu et al. [16] investigated natural convection heat transfer around a radial heat sink adapted for dissipating heat on a circular LED, and found that as the number of surface patterns increased, the thermal resistance and average heat transfer coefficient decreased. Micheli et al. [17] fabricated micro-finned arrays on wafer through a dicing machine and investigated the effects of fin geometry, orientation and materials on heat transfer properties. Results showed that the heat transfer coefficient increases with the spacing increase and the height decrease. Liu et al. [18] studied the effect of five kinds of microstructure geometries on forced convection heat transfer, and found that the average Nusselt number of V-shaped grooved microstructure could be increased by about 1.6 times. Sun et al. [19] fabricated V-shape microgrooves on polypropylene (PP) layer by isothermal hot embossing, and fabricated an aluminum-PP coupled heat sink with heat dissipation rate around 2.5%. Hung et al. [20] investigated the effects of substrate materials, coolants, and geometric parameters on temperature distribution and thermal resistance of the double-layered microchannel heat sink, showing an average increasing of 6.3% of thermal performance. Kim et al. [21] fabricated micron size arrays on silicon wafers with microelectromechanical systems (MEMS) technique, and showed that the enhancement of heat dissipation was at most 10%. Zhuang et al. [22] established a metal–polymer microstructure heat sink model, and showed that the total thermal resistance could be reduced by almost 29.9%. However, most of surface structures were fabricated on either Si or metal substrate, which limited further application in flexible electronic devices. Moreover, the traditional processing method for flexible substrate restricts the accuracy of surface structures, thus limiting heat dissipation performance. 

In this work, metal-polymer composite heat sink with textured PET was designed, and natural convection heat transfer properties were studied. Micro-groove and micro-grid with depth range from 10 to 50 μm and spacing range from 100 to 250 μm were fabricated on PET substrate through UV laser. The temperature evolution was investigated experimentally and numerically. Our results offer a new lightweight and effective metal-polymer composite heat sink, which is an alternative of large and heavy metal coolers.

## 2. Materials and Methods 

### 2.1. Materials

The 100-μm-thick polyethylene terephthalate (PET) was obtained from DuPont (Shanghai, China). The 200-μm-thick Al6061 was from Alcoa (New York, NY, USA). The LED chip was achieved from BOSMFC Optoelectronics Co. Ltd. (Dongguan, China). The silicone heater was from Fullchance Industrial Co., Ltd. (Shenzhen, China).

### 2.2. Laser Surface Texturing

The laser surface texturing was applied on the PET tape with a wavelength of 354 nm solid-state laser. The laser system was equipped with a laser head and an F-Theta focusing lens allowing the delivery of a focused laser beam over the sample surface with beam spot diameter of 40 μm. Laser pulse duration of 15 ns, average power of 5 W and repetition rate of 100 kHz were used for all texturing processes.

**Figure 1:**
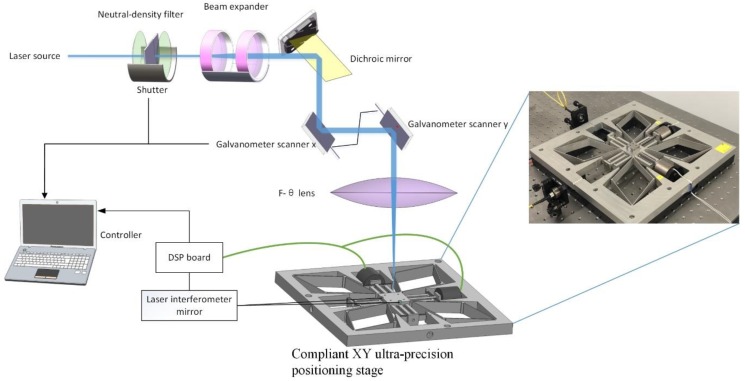
Schematic of experimental setup for laser surface texturing using an ultra-precision stage.

A flexure-based XY ultra-precision stage is utilized to fabricate precise features and achieve high repeatability at the surface of workpiece due to its advantages of frictionless bearings, no assembly and zero maintenance [23]. As shown in Figure 1, the stage is based on a parallel kinematic configuration of the flexure mechanism, so that the uniform high bandwidth of each axial motion can be achieved. During laser surface texturing, the stage is mounted on a floatation platform for vibration suppression purpose. An Attocube (Muenchen, Germany) laser interferometer is utilized for real-time displacement feedback, while a real-time feedback controller is developed and implemented on the above stage. The stage moves according to the command reference, correspondingly, the workpiece moves in a planar motion.

Micro-groove and micro-grid on the PET is performed by scanning the surface with the laser beam with the above stage in motion. Textures were produced with fixed distance ranging from 100 μm to 250 μm and with the depth ranging from 10 μm to 50 μm.

### 2.3. Experimental Setup

**Figure 2:**
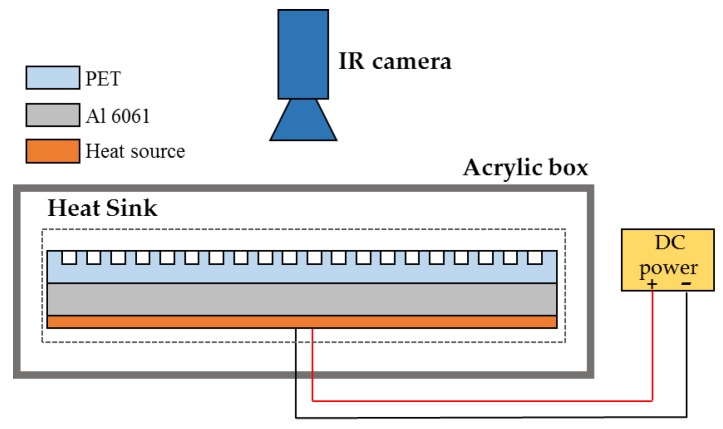
Schematic of the experimental apparatus.

The schematic of the experimental apparatus is shown in Figure 2, where a metal-polymer composite heat sink is used as the cooling unit, and a silicone heater with a DC power is used as the heat source unit. The metal-polymer composite heat sink consists of a 20 × 15 × 0.2 mm^3^ Al 6061 metal used as heat conducting panel and a 20 × 15 × 0.1 mm^3^ laser surface textured PET used as the dissipation unit. The PET-based heat sink is placed in the acrylic box to prevent interference from surroundings. A high speed IR camera is used for temperature measurement and temperature field visualization.

Experiments for heat dissipation capability is conducted as follows: first, the silicone heater is used to heat the PET-based heat sink. Then, turn off the DC power when the temperature of PET exceeded 85 °C. At last, let the PET dissipate heat under steady state natural convection conditions and record the time taken to drop ever 10 °C from 80 °C to 20 °C.

### 2.4. Numerical Models and Governing Equations

Table 1 shows the different material parameters of each layer of heat sink. CFD models of metal-plastic composite heat sink have been generated in FloEFD^®^ (Mentor Graphics, Wilsonville, OR, USA) to explore the thermal performances. In the simulation, the external boundary condition with the inlet pressure is set as 1 atm. The ambient temperature is set as 20 °C. The heat source is a surface source where the heat generation rate is set as 0.5 W. 

To conduct the analysis, we make the following assumptions [22]:

(1) The solid materials are isotropic;
(2) The heat transfer at each surface exposed to the air is governed by natural convection;
(3) The contact thermal resistance between contact areas is neglected;
(4) The bottom of heat source is adiabatic.

Based on Fourier’s law and energy conservation, the analysis is conducted as follows [24]:

For the heat sink, when the heat balance is reached, the entire heat transfer through the metal-plastic composite heat sink satisfies the following equation:

(1) $$Q\;=\;-\lambda_{1}A_{1}\frac{T_{2}-T_{1}}{\delta_{1}}$$

The following equation holds for the heat transfer from the heat source to the metal:

(2) $$-\lambda_{1}A_{1}\frac{T_{2}-T_{1}}{\delta_{1}}\;=\;-\lambda_{2}A_{1}\frac{T_{3}-T_{2}}{\delta_{2}}$$

The heat transfer from the metal to PET is described by the following equation:

(3) $$-\lambda_{2}A_{1}\frac{T_{3}-T_{2}}{\delta_{2}}\;=\;-\lambda_{3}A_{1}\frac{T_{4}-T_{3}}{\delta_{3}}$$

Finally, the calculation of heat transfer from the PET layer to the environment is as follows:

(4) $$-\lambda_{3}A_{1}\frac{T_{4}-T_{3}}{\delta_{3}}{\;=\;\mathrm{hA}}_{2}\left(T_{4}-T_{f}\right){\;+\;A}_{2}\varepsilon \sigma \left(T_{4}^{4}-T_{f}^{4}\right)$$

where Q is the entire heat loss through the metal-plastic composite heat sink, and λ_1_, λ_2_ and λ_3_ are the thermal conductivity of heat source, conducting metal and PET, respectively, and δ_1_, δ_2_ and δ_3_ are the thickness of heat source, conducting metal and PET, respectively, and T_1_, T_2_, T_3_ and T_4_ are the surface contact temperature of heat source, conducting metal, bottom of PET and top of PET, respectively, and σ is the constant of black-body radiation, and ε is the radiant emissivity of PET, and T_f_ is the ambient temperature, and A_1_ and A_2_ is the bottom area of metal substrate and total radiation area of textured PET, respectively. The parameters used in the above equations are listed in Table 2.

### 2.5. Analysis and Characterization

The topographies of laser textured surfaces are measured by an optical microscope (OM, LV150N, Nikon, Tokyo, Japan) and a 3D laser scanning confocal microscope (VK100, Keyence, Osaka, Japan). A high-speed IR camera (E95, FLIR, Wilsonville, USA) is used for temperature field visualization and measurements. A scanning electron microscope (SEM, Quanta 450 FEG, FEI, Hillsboro, OR, USA) is utilized to observe the textured surface of PET.

## 3. Results and Discussion

### 3.1. Surface Morphology

**Figure 3:**
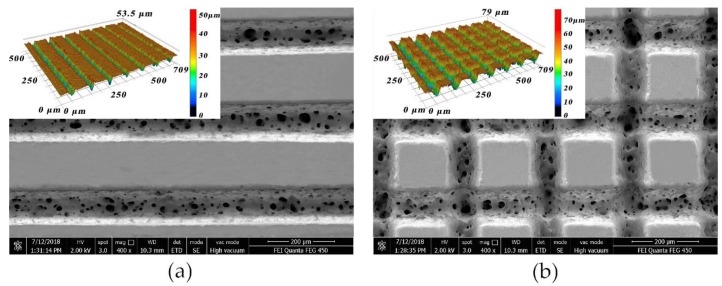
Surface morphology of the laser textured surfaces: (**a**) micro-groove; (**b**) micro-grid.

Micro-groove and micro-grid with spacing from 100 μm to 250 μm and depth from 10 μm to 50 μm were fabricated on the PET substrate. The typical topography of the fabricated micro-groove and micro-grid is shown in Figure 3. It is worth noting that craters with the diameter ranging from 4.5 µm to 30 µm have been formed along laser scanning tracks. During laser processing of PET, a two-phase liquid-gas mixture developed after thermalization of the laser energy in the material [25]. Due to subsurface heating effects, materials inside the PET were molten, resulting in vapour bubbles on basis of polymer decomposition as CO_2_ and CO [26]. The gas inside these bubbles was thermally expanded until thin wall of the bubbles can no longer hold the raising pressure and break. The gas passed through the molten region and ejected from the bulk to the outside surface of the PET once the bubbles exploded, resulting in formation of craters. This is in good agreement with polymethyl methacrylate craters with the diameter ranging from 5 µm to 20 µm produced by nanosecond UV laser according to the findings of Efthimiopoulos [27] and Britta [28].

### 3.2. Dependence of the Heat Dissipation Performance on the Textured PET Heat Sink

**Figure 4:**
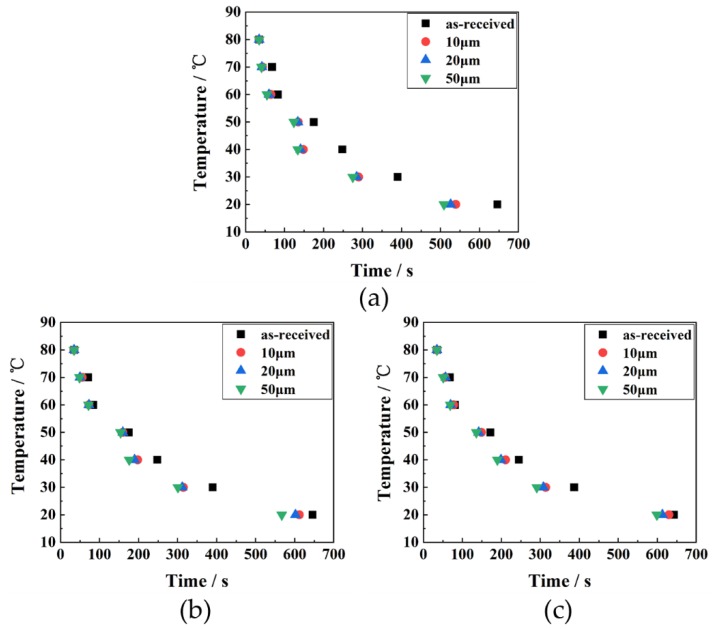
The cooling time of micro-groove for different height with the spacing of: (**a**) 100 μm; (**b**) 150 μm; (**c**) 250 μm.

The PET temperature variation with respect to time for as-received and micro-grooved PET-based heat sink is shown in Figure 4a–c. It can be seen that all micro-grooved PET-based heat sink has higher heat dissipation efficiency than that of as-received specimens. With the decrease of temperature difference, the effect of micro-groove on heat dissipation becomes more significant. As shown in Figure 4a–c, for the fixed spacing of micro-groove (100, 150 and 250 μm), the cooling time is inverse proportional to the micro-groove depth, because the thermal resistance is inverse proportional to the micro-groove depth, resulting in enhanced heat dissipation [29]. The micro-holes formed along the micro-groove lead to surface area increase [30] and small pressure drop [31]. The small pressure drop could result in a higher air velocity, which is useful for enhancing heat dissipation efficiency [32]. Also, it is observed that for fixed micro-groove depth (10, 20 and 50 μm), the cooling time is proportional to the micro-groove spacing, which is mainly due to the increased texture spacing leading to surface area reduction, resulting in the decrease of dissipation efficiency and increase of thermal resistance [33].

**Figure 5:**
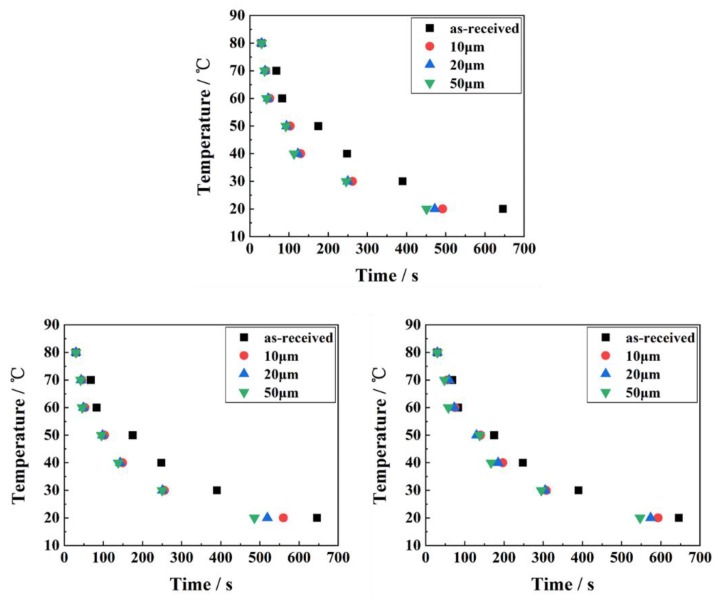
The cooling time of micro-grid for different height with the spacing of: (**a**) 100 μm; (**b**) 150 μm; (**c**) 250 μm.

The temperature variation with respect to time for as-received and micro-grooved heat sink is shown in Figure 5a–c. The cooling time curve of the micro-gridded heat sink is similar to that of the micro-grooved one in Figure 4a–c, yet with smaller value. 

A summary of the heat dissipation performance for each test specimen is given in Table 3, where the heat sink with as-received PET takes the longest time (about 593 s) to reduce the temperature from 80 °C to 20 °C. The total cooling time decreases with the increase of texture depth and decrease of texture spacing. For a fixed textured depth, the reduced texture spacing leads to more surface area for heat dissipation and smaller unit size, resulting in a faster cooling rate. Therefore, the total cooling time is proportional to the spacing width for a fixed texture depth. Meanwhile, for a fixed texture spacing, the increased texture depth leads to more area available for convection, resulting in thermal resistance decreases. Therefore, the total cooling time is inversely proportional to the spacing depth for a fixed textured spacing. For the same geometric parameters with a depth of 50 μm and spacing of 100 μm, the total cooling time of micro-grooved and micro-gridded heat sinks is 475 s and 428 s, respectively, while that of the as-received is 593 s. 

The heat transfer coefficient can be obtained from the measured temperature difference and the power input by the following equation [21]:

(5) $$h\;=\;\frac{Q_{\mathrm{in}}}{A_{t}\left(T_{P}-T_{\infty }\right)}$$

where h is the heat transfer coefficient of the textured PET, and Q_in_, T_P_ and T_∞_ are the input power, textured PET temperature and ambient temperature, respectively, and A_t_ is the total surface area of textured PET.

Table 3 also shows the total heat transfer coefficient at temperature difference of 20 °C. It is seen that the as-received PET has the highest heat transfer coefficient (11.51 Wm^−2^K), which is consistent with the previous findings [34], because the heat transfer coefficients is inverse proportional to the total surface area. It is worth noting that the heat transfer coefficient of micro-grid is lower than that of micro-groove, because the micro-grid has a larger surface area than that of micro-groove. Micro-grid with a depth 50 μm of and a spacing of 100 μm has the lowest heat transfer coefficient, and it is proportional to the texture spacing. It can be seen from Table 3 that the heat dissipation behavior is dependent of heat transfer coefficient. Note that if the temperature difference in Equation (5) is fixed, the product of heat transfer coefficient and total area can be used to evaluate the texture effectiveness by the following equation [21]:

(6) $$e=\frac{Q_{\mathrm{in}1}}{Q_{\mathrm{in}2}}=\frac{h_{p}A_{p}}{h_{a}A_{a}}$$

where Q_in1_, h_p_ and A_p_ are the input power, heat transfer coefficients and of textured specimen respectively, and Q_in2_, h_a_ and A_a_ are the input power heat transfer coefficients and total surface area of as-received specimen, respectively. From Table 3, the texture effectiveness of micro-gridded PET with depth of 50 μm and spacing of 100 μm is 25% better than that of the as-received PET.

### 3.3. Cooling Performance of LED Chip with Textured PET Heat Sink

**Figure 6:**
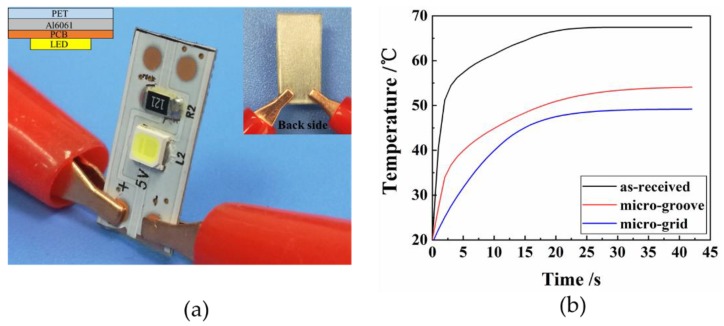
(**a**) The physical map of the heat cooling device; (**b**) Transient temperature of the center temperature of the LED chip for micro-groove and micro-grid.

To verify cooling performance of the laser-textured PET heat sink, three types of natural convection cooling devices for LED chips were produced on basis of as-received, micro-grooved and micro-gridded PET with a depth of 50 μm and a spacing of 100 μm, as shown in Figure 6a. When voltage was applied, the chip temperature raised and tended to be stable over time. The temperature rising curves of these cooling device are shown in the Figure 6b. The heat dissipation process of LED chips can be divided into two stages: unsteady stage and steady stage. At the initial unsteady stage, the heat generated by electric current led to the increase of LED chip temperature. The heat was mainly conducted to the heat sink side and then dissipated to air. When heat dissipation capacity of LED chip increased, it required a longer time to reach equilibrium temperature. Therefore, the heat sink with as-received PET led to a significant increase of the LED chip temperature due to poor heat dissipation capacity. As the result of heat transfer, the balance between heat generation and heat dissipation was achieved, leading to equilibrium temperature of the LED chip. The temperature rising curves shown in the Figure 6b indicated that the equilibrium of LED chip temperature with as-received, micro-grooved and micro-gridded were 64.7 °C, 53.6 °C and 50.2 °C, respectively. We propose that the micro-gridded LED has the optimized heat dissipation performances rather than the others. The results are in agreement with the heat dissipation test in Section 3.2.

**Figure 7:**
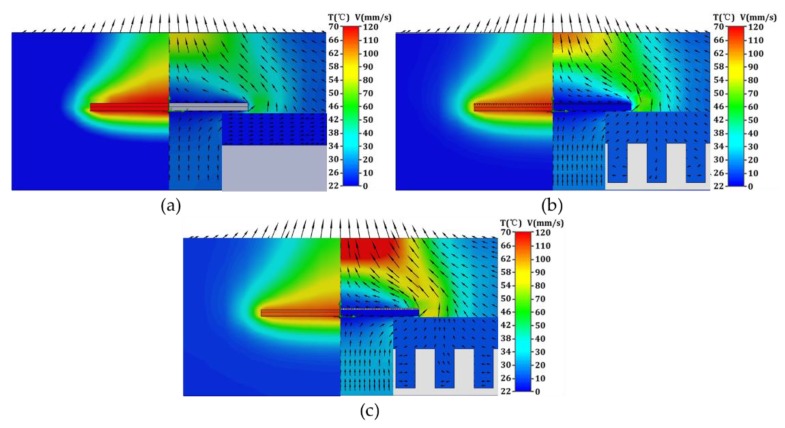
Temperature field and Air velocity field around the heat cooling devices: (**a**) as-received; (**b**) micro-groove; (**c**) micro-grid.

Finite element analysis (FEA) was further employed to analyze the heat propagation process of the above three LED chips. Figure 7 illustrates both the velocity and temperature fields of as-received, micro-grooved and micro-gridded LED, and the inset is the velocity field between micron-size features. It is shown that the flow patterns of the three chips are single chimney type flow pattern, which was firstly observed by Harahap [35]. There exists a main upward airflow with cold air flow from the bottom to upward and almost stationary near the heat sink [36]. However, the velocity distribution of these chips is different. As shown in Figure 7a, the existence of large low velocity area implies that the airflow is blockage and the heat transfer area is not adequate, resulting in the poorest heat dissipation performance. Figure 7b shows a much higher velocity around the micro-grooved heat sink, indicating a better natural convection, which enhances the heat dissipation performance in turn. Figure 7c shows that the maximum air velocity of micro-gridded PET can be obtained as 120 mm/s along the central line with further enhanced velocity distribution, which is mainly attributed to the minimized negative effect of stationary air on heat dissipation performance.

**Figure 8:**
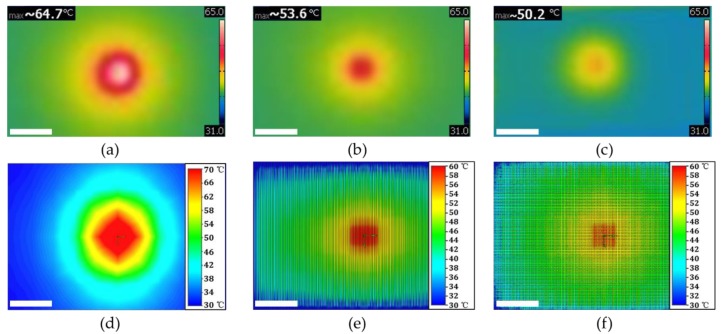
Steady-state temperature fields of: (**a**) as-received; (**b**) micro-groove; (**c**) micro-grid. (**d**–**f**) are the corresponding simulation results. The scale bar is 3 mm.

Figure 8a–c gives the steady-state temperature fields of three LED chips. Regions where the contours are white and red indicate the high temperature, while the blue contours represent the low value. As shown in Figure 8a, a large high-temperature area occurs at the center LED chip with as-received PET, while only small high-temperature area occurs at the center of micro-grooved PET (Figure 8b) and micro-gridded PET (Figure 8c). LED with the as-received PET shows the highest temperature 64.7 °C compared to micro-groove with 56.3 °C and micro-grid with 50.2 °C. The smaller high-temperature area and lower LED chip temperature indicate that the textured PET provides sufficient thermal conductive ability to spread and remove heat from the LED chip [37]. Figure 8d–f shows the corresponding simulated temperature fields of three PET substrates. Simulation results show that lowest LED chip temperature is achieved with micro-gridded PET. The results are well in accord with the experimental results. However, it should be noticed that the value of LED chip temperature estimated by simulation is a little larger than those obtained from experimental results. This is probably because of the difference between the boundary conditions of simulation and the experiment [38].

## 4. Conclusions

A metal-plastic composite heat sink with laser surface textured PET served as a cooling unit is proposed. Convection heat transfer experiment was carried out to characterize thermal performance of three types of heat sinks, i.e., as-received, micro-grooved and micro-gridded PET. The heat dissipation performance of the LED chip cooling device fabricated with two optimized heat sinks was investigated experimentally and numerically. The main conclusions are listed as following:

1. Smaller texture spacing and larger texture depth were shown to be beneficial to reduce thermal resistance, resulting in increase of dissipation efficiency.
2. Micro-grid with a depth of 50 μm and a spacing of 100 μm enhances the effectiveness by 25% and reduces cooling time by 27.8%. This is mainly due to the combined effects of large surface area, low thermal resistance and natural convection reinforcement.
3. FEA results show that the heat cooling devices with textured PET can significantly improve air velocity distribution and a more effective heat transfer to the air, resulting in the enhanced heat dissipation performance.
4. Compared to the LED chip with as-received PET substrate, the LED chip with optimized laser-textured PET led to the temperature reduction by 22.4%.

## Figures and Tables

**Table 1:** Physical parameters of the different materials.

Material	Density (g/cm^3^)	Specifc Heat (J/g⋅K)	Termal Conductivity (W/m⋅K)	Types of Thermal Conductivity	Melting Temperature (K)
Air	1.21	1.01	0.03	Isotropic	-
Al6061	2688.90	0.90	237	Isotropic	855
PET	1.35	1.28	0.28	Isotropic	520

**Table 2:** Parameters in Equation (1) to Equation (4) for simulation.

Thermal Conductivity (W/m⋅K)			Thickness (um)			Radiant Emissivity (ε)
λ_1_	λ_1_	λ_1_	δ_1_	δ_2_	δ_3_	ε
381	237	0.28	50	100	100	0.7

**Table 3:** Total cooling time, heat transfer coefficient and fin effectiveness of specimen.

Specimen	Depth (μm)	Spacing (μm)	Total Cooling Time (s)	Heat Transfer Coefficient (Wm^−2^K)	Texture Effectiveness
As-received	/	/	593	11.51	1
		100	499	9.62	1.02
	10	200	564	10.1	1.02
		250	589	10.51	1.01
		100	489	8.91	1.10
Micro-groove	20	200	553	9.71	1.09
		250	578	10.12	1.04
		100	475	7.81	1.19
	50	200	517	8.12	1.18
		250	559	9.18	1.13
		100	464	9.93	1.05
	10	200	531	10.01	1.04
		250	553	10.32	1.03
		100	446	9.41	1.16
Micro-groove	20	200	492	9.64	1.15
		250	534	9.82	1.13
		100	428	7.14	1.25
	50	200	461	7.30	1.20
		250	517	8.12	1.16
